# Dynamic coronal plane knee alignment: Femoral anatomy determines kinematic curve morphology, tibial anatomy determines curve position

**DOI:** 10.1002/ksa.70152

**Published:** 2025-11-05

**Authors:** Petros Ismailidis, Anthony Leicht, Kenji Doma, Peter McEwen

**Affiliations:** ^1^ The Orthopaedic Research Institute of Queensland (ORIQL) Pimlico Queensland Australia; ^2^ Sport and Excercise Science James Cook University Douglas Queensland Australia; ^3^ Department of Orthopaedics and Traumatology University Hospital of Basel Basel Switzerland; ^4^ The Australian Institute of Tropical Health and Medicine James Cook University Douglas Queensland Australia

**Keywords:** dynamic alignment, HKA, kinematic curve, navigation, robotic, TKA

## Abstract

**Purpose:**

A single hip–knee–ankle angle (HKA) angle does not reflect the biomechanics of native, arthritic or prosthetic knees. Since HKA varies throughout flexion, dynamic coronal alignment is best represented by a kinematic curve plotting HKA against the range of motion. This study aimed to evaluate the relationship between kinematic HKA curves and the bony morphology of the distal femur and proximal tibia. We hypothesised that variations in distal femoral and proximal tibial anatomy are associated with distinct patterns of dynamic coronal alignment.

**Methods:**

This was an experimental study using a non‐weight‐bearing articulated surgical education bone model including hemipelvis, femur and tibia. Articular surfaces and bony landmarks were registered with a computer navigation system. Using medial opening wedge femoral and tibial osteotomies and a rotational femoral osteotomy, 70 morphotypes were created by altering distal femoral angle (DFA), proximal tibial angle (PTA) and femoral axial angle (FAA). For each configuration, a coronal kinematic curve was recorded from 0° to 120° of flexion.

**Results:**

Five curve morphotypes were identified: straight, drift, inverse drift, C‐shaped and inverse C‐shaped. DFA and FAA differed significantly among morphotypes (*p* < 0.001), whereas PTA had no effect (*p* = 0.084). Paired comparisons confirmed significant differences in DFA and FAA across curve types.

**Conclusion:**

In this sawbone model, dynamic coronal plane alignment curve morphology was determined by distal femoral coronal and torsional anatomy, while tibial anatomy shifted the curve position without altering morphology. Restoring the pre‐arthritic curve in TKA requires restoring DFA and FAA, whereas achieving a neutral straight curve requires individualised FAA adjustment. Consistently producing a neutral straight curve is not possible without computer‐assisted or robotic surgery. These findings require validation in cadaveric or clinical studies but may guide surgical strategies aiming to reproduce native knee kinematics.

**Level of Evidence:**

N/A.

**Abbreviations**
DFA: distal femoral angle
FAA: femoral axial angle
HKA: hip–knee–ankle angle
KATKA: kinematically aligned TKA
MATKA: mechanically aligned TKA
PCA: posterior condylar axis
PTA: proximal tibial angle
STEA: surgical transepicondylar axis
TKA: total knee arthroplasty

## INTRODUCTION

**Figure 1:**
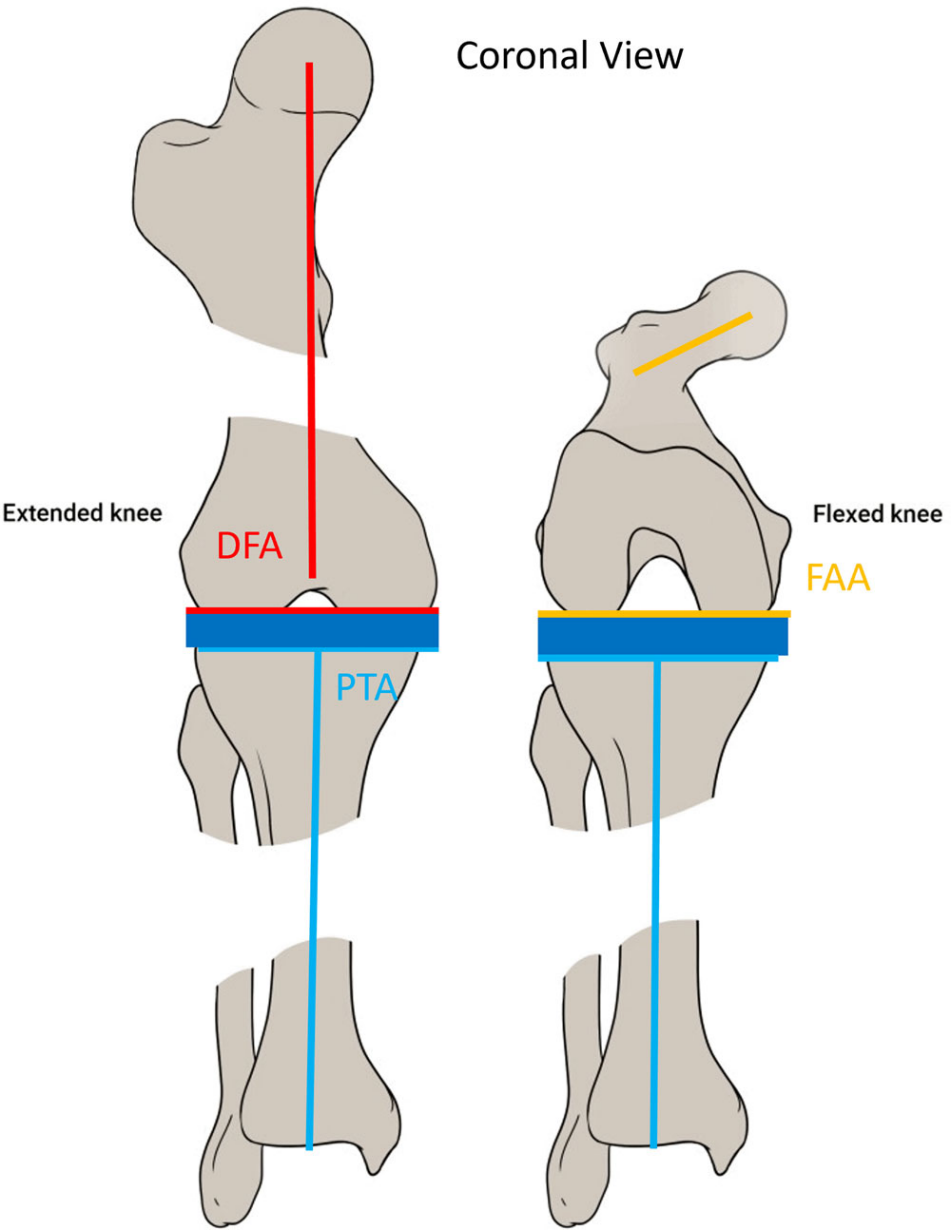
Coronal knee alignment bone cuts during total knee arthroplasty in extension (left) and flexion (right). The orientation of the bone cuts defines the component placement. The distal femoral angle (DFA, red) and proximal tibial angle (PTA, blue) of the bone cuts define the coronal knee alignment in extension. The femoral axial angle (FAA, yellow) and PTA of the bone cuts define the knee alignment in flexion.

A large body of total knee arthroplasty (TKA) research has focused on constitutional alignment and the corresponding placement of the femoral and tibial implants in the coronal and axial plane to restore constitutional alignment [10, 14]. The hip–knee–ankle angle (HKA) describes the alignment of the knee in the coronal plane. The HKA in full extension is defined by the distal femoral angle (DFA) and the proximal tibial angle (PTA) [13], while the coronal alignment in 90° flexion (‘HKA’ in flexion) is defined by the femoral axial angle (FAA) and the PTA of the prosthetic components (Figure 1). In flexion, load transmission shifts to the posterior femoral condyles. In this position, axial rotation of the femur directly alters which condylar region articulates with the tibia. Internal femoral rotation elevates the lateral condyle, effectively producing a valgus HKA in flexion, whereas external femoral rotation elevates the medial condyle, resulting in a varus HKA in flexion [2].

Mechanically aligned TKA (MATKA) aligns both the femoral and tibial components perpendicular to the mechanical axis of the limb to achieve neutral alignment in extension, in other words, a HKA, DFA and PTA of 0°. Femoral component rotation is related to any of a number of femoral landmarks or by gap balancing the flexion gap [9, 10]. The effect of femoral component rotation on dynamic HKA is generally not considered. The aim of kinematically aligned TKA (KATKA) is to restore the pre‐arthritic HKA by placing the tibial and femoral components according to the individual's pre‐arthritic PTA, DFA and posterior condylar axis (PCA) [6].

Recent research has demonstrated that the idea of a single HKA throughout the range of motion does not reflect the biomechanics of native, arthritic knee joints or TKAs [3, 7, 12, 16, 17, 18, 20] with the HKA varying through the flexion arc. Therefore, coronal knee alignment is dynamic and best described through a graphic presenting a curve of HKA angles through the range of motion rather than through a single HKA value. Computer navigation and robotic systems are gaining popularity in TKA [4] because of the increased precision in component placement, which they provide [5, 15]. These systems can deliver detailed multidimensional kinematic information, which can be presented in the form of a coronal plane kinematic curve through the range of motion.

**Figure 2:**
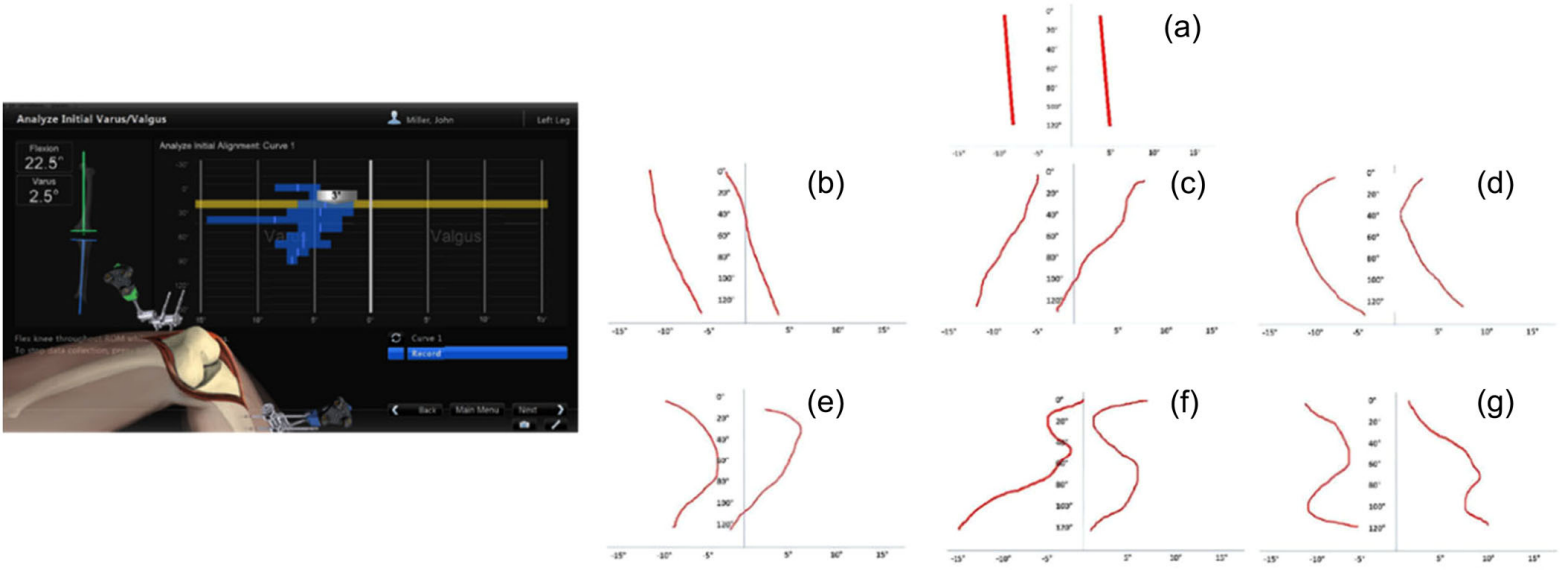
The hip–knee–ankle curve through the range of motion as measured by computer navigation systems (left, OrthoMap® Precision Knee, Stryker Navigation, MI 49001, USA). Curves are plotted to illustrate coronal plane angles on the *x*‐axis (‐ denotes a varus angle) and the flexion on the *y*‐axis. The seven different individual coronal kinematic knee curve morphotypes defined by Young et al. [20], namely straight (a), drift (b), inverse drift (c), C‐shaped (d), inverse C‐shaped (e), S‐shaped (f) and inverse S‐shaped (g) (right).

Various studies have examined the dynamic knee alignment and discussed the fact that the HKA changes through flexion [3, 7, 12, 16, 17, 18, 20]. Young et al. [20] used principal component analysis of the pre‐ and post‐TKA kinematic curves of a large cohort of arthritic knees treated with computer‐navigated TKA to classify individual curve morphotypes. They identified four principal component curves: straight, oblique, drift, C‐shaped and S‐shaped. Apart from the straight type, each of the other curves additionally had a reverse variant making a total of seven curve types (Figure 2). To date, no study has identified the factors driving curve morphology.

It is currently unknown whether TKA will function best by replicating the pre‐arthritic dynamic knee alignment by replicating the native pre‐arthritic curve morphotype or by altering it to a neutral coronal curve. Regardless, it is impossible to reliably restore or alter an individual's curve morphotype without understanding what determines curve morphology in the first place. Therefore, we conducted a study aimed at evaluating the relationship between kinematic HKA curves across the range of motion and the underlying bony morphology of the distal femur and proximal tibia.

We hypothesised that the DFA, PTA and FAA would have a significant correlation with the kinematic curve morphology.

## MATERIALS AND METHODS

### Ethics and registration

The study did not require Human Research Ethics Committee approval, as it was conducted using a synthetic bone model and involved no human participants or data.

### Procedures

**Figure 3:**
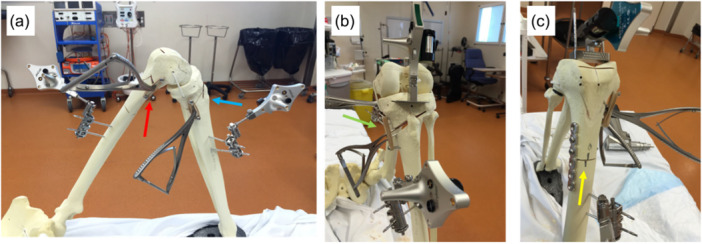
The articulated lower limb surgical education bone model consisting of hemipelvis, femur and tibia with optical navigation arrays attached. (a) Medial opening wedge femoral osteotomy (red arrow) and tibial osteotomy (blue arrow). (b) Distractor in the tibial osteotomy in place (green arrow). (c) Rotational femoral osteotomy showing the torsional zero reference line (yellow arrow).

An articulated lower limb surgical education bone model consisting of hemipelvis, femur and tibia was used (Figure 3). In its unaltered state, the model exhibited an HKA angle of 8° varus and 2° of femoral antetorsion. The knee joint was stabilised using cords to simulate both collateral and cruciate ligaments.

Limb registration was performed with a computer navigation system (OrthoMap® Precision Knee, Stryker Navigation, MI, USA) following the manufacturer's instructions (Precision pathway). Briefly, the femoral mechanical axis was defined as the straight line from the hip centre to the knee centre. The hip centre was determined functionally by pivoting the femur under navigation to identify the centre of the femoral head, while the knee centre was calculated as the midpoint between the medial and lateral femoral epicondyles. For the tibial mechanical axis, the ankle centre was defined as the midpoint between the medial and lateral malleoli, and the axis was defined as a straight line from the knee centre to the ankle centre. The PCA and the surgical transepicondylar axis (STEA) were colinear in the reference model. The PCA was used to define the axial plane, with coronal and sagittal planes defined orthogonally to this reference.

Coronal orientations of the distal femur and proximal tibia were then measured and recorded. The HKA was defined as the angle between the mechanical axis of the femur and that of the tibia. The DFA (positive values = valgus) was defined as the angle between the mechanical femoral axis and the distal femoral joint line. The PTA (positive values = valgus) was defined as the angle between the mechanical tibial axis and the proximal tibial joint line. The FAA (positive values = external rotation) was defined as the angle between the PCA and the femoral neck axis [13] (Figure 1).

For each configuration, a dynamic coronal kinematic curve was obtained by manually moving the model through a flexion range of 0°–120°, unloaded but ensuring constant medial and lateral articular contact. Relative tibiofemoral rotation was also recorded. If relative rotation exceeded 10° throughout the flexion arc, the measurement was repeated until a consistent and satisfactory curve was acquired.

Three osteotomies were performed to create knee joints with varying combinations of DFA, PTA and FAA, representing a wide spectrum of normal femoral and tibial anatomy, including outliers. This approach ensured that all coronal plane alignment types (CPAKs) [8] were included in the study.

- A medial opening wedge femoral osteotomy: A step cut was created to prevent anteroposterior and rotational instability. A screw was placed in the medial cortex either side of the osteotomy, and a tensioned rubber band was mounted between the screw heads to provide compression across the osteotomy. Using a laminar spreader, the osteotomy was distracted to vary DFA (Figure 3a and Table 1). Each incremental change in DFA represented one click on the laminar distractor ratchet. After each adjustment, the distal femoral anatomy was re‐registered with the computer navigation system to document the DFA of the ‘new femur’ following distraction.
- A rotational femoral osteotomy: A straight line parallel to the anatomical femoral axis was marked longitudinally on the anterior cortex of the femur at the junction of the middle and distal third as a zero reference. A rotational osteotomy was performed perpendicular to this line. The osteotomy was stabilised with a dowel rod press fit into the reamed intramedullary canal and a dynamic compression plate. The rotational osteotomy was used to vary FAA (Figure 3c and Table 1). FAA was adjusted by flexing the knee 90° and manually adjusting femoral torsion with a validation tracker inserted into a slot cut into the distal femur parallel to the STEA.
- A medial opening wedge tibial osteotomy: A step cut was created to prevent AP and rotational instability. The same screw and band construct used on the femur was applied to the tibial osteotomy. Using a laminar spreader, the osteotomy was distracted to vary PTA (Figure 3a,b and Table 1). Each incremental change in PTA represented one click on the laminar distractor ratchet. After each adjustment, the proximal tibia anatomy was re‐registered with the computer navigation system to document the PTA of the ‘new tibia’ following distraction.

**Table 1:** Curve groups and distal femoral angle (DFA), proximal tibial angle (PTA) and femoral a angle (FAA) for each group.

Curve group	DFA	FAA	PTA
1	Fixed at 0°	Fixed at 0°	Varied −6.5° to 3°
2	Varied −1.5° to 7.5°	Fixed at 0°	Fixed at 0°
3	Fixed at 0° (neutral)	Varied −10° to 10°	Fixed at 0°
4	Fixed at 1.5° (modal anatomy)	Varied −10° to 10°	Fixed at −3°
5	Fixed at 4.5° (joint obliquity outlier)	Varied −10° to 10°	Fixed at −5.5°
6	Fixed at −1.5° (varus outlier)	Varied −10° to 10°	Fixed at −5.5°
7	Fixed at 6° (valgus outlier)	Varied −10° to 10°	Fixed at 1°

*Note*: A positive value denotes valgus/external torsion. ‘Neutral’: Represents straight knees with HKA, DFA and PTA of 0°. ‘Modal anatomy’: Represents average knees with valgus DFA and varus PTA within the normal population range. ‘Joint obliquity outlier’: Represents a combination of valgus DFA of 4.5° and varus PTA of –5.5°, resulting in an arithmetic HKA of –1° (within the normal range) but with increased joint line obliquity. ‘Varus outlier’: Represents varus knees with DFA in extreme varus outside the normal range (0°–5°) combined with PTA in varus outside the normal range (–5° to 0°). ‘Valgus outlier’: Represents knees with DFA in extreme valgus outside the normal range (0°–5°) combined with PTA in valgus outside the normal range (−5° to 0°).

Curves for 70 different morphotypes were recorded by sequentially altering one variable in each group (Table 1).

**Figure 4:**
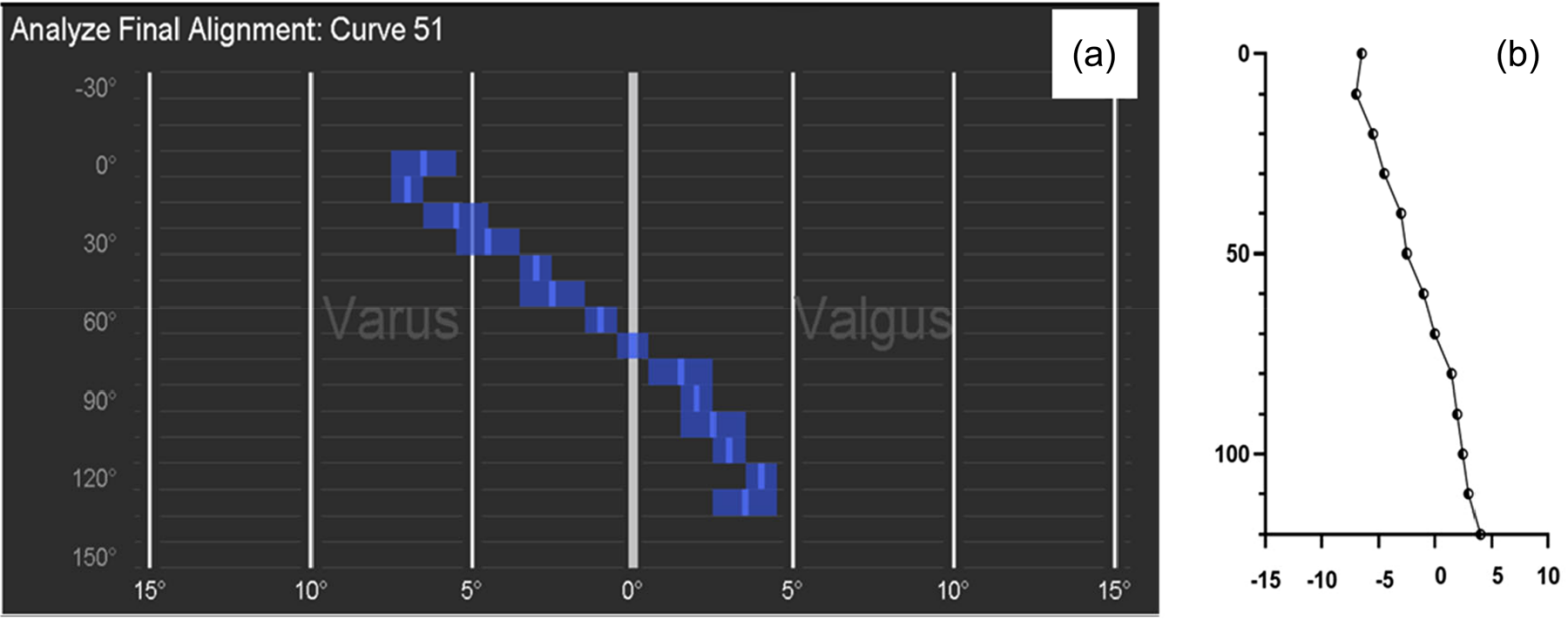
(a) Hip–knee–ankle angle (HKA) graphic from navigation system. (b) Line scatter plot generated from (a) showing the HKA on the *x*‐axis (‐ denotes varus) and the knee flexion on the *y*‐axis. This graph shows a knee with a ‘drift’ kinematic curve with an HKAA of 6° varus in extension, 0° HKAA in 70° flexion and 4 ° of valgus in 120° flexion.

Each kinematic curve graphic was exported from the computer navigation program and hyperlinked to its coordinate combination in a digital spreadsheet. The light blue bar at each flexion increment was recorded as the HKA, and a subsequent line scatter plot representing HKA through flexion range was produced (Figure 4). Each scatter plot was classified into a curve morphotype according to the seven types described from Young et al. [20] as defined in Table 2.

**Table 2:** Curve morphotype definitions are based on the hip–knee–ankle angle (HKA) deviation in the line scatter plot.

Curve type	Definition
Straight	<3° deviation of HKA between 0° and 120° of flexion
Drift	Deviation of HKA values in a valgus direction ≥3° between 0° and 120° of flexion
Inverse drift	Deviation of HKA values in varus direction ≥3°
C‐shaped	Parabolic curve with the apex towards varus (initial HKA deviation towards varus of ≥3° followed by a later HKA deviation back towards valgus of ≥3° between 0° and 120° of flexion)
Inverse C‐shaped	Parabolic curve with the apex towards valgus (initial HKA deviation towards valgus of ≥3° followed by a later HKA deviation back towards varus of ≥3° between 0° and 120° of flexion)
S‐shaped	Compound C‐shaped followed by inverse C‐shaped
Inverse S‐shaped	Compound inverse C‐shaped followed by C‐shaped

*Note*: Each curve is formed by connecting 13 points, corresponding to 13 HKA values. These HKA values are then used to classify the curve into the different curve types, as described above.

### Statistical analysis

Statistical analysis and graph preparation were performed using Graphpad Prism 9.0 for Windows (GraphPad Software, San Diego, California, USA). Continuous data were tested for normality with the D'Agostino and Pearson test. Statistical comparison of central tendencies was subsequently performed using the Kruskal–Wallis test with multiple comparisons using Dunn's multiple comparison test (due to non‐normality of data). Magnitude of difference or effect size was determined from the eta squared estimate (*ε* ^2^) of the Kruskal–Wallis test with the interpretation as follows: 0.010–0.059 (small effect), 0.06–0.139 (moderate effect) and ≥0.14 (large effect).

## RESULTS

**Figure 5:**
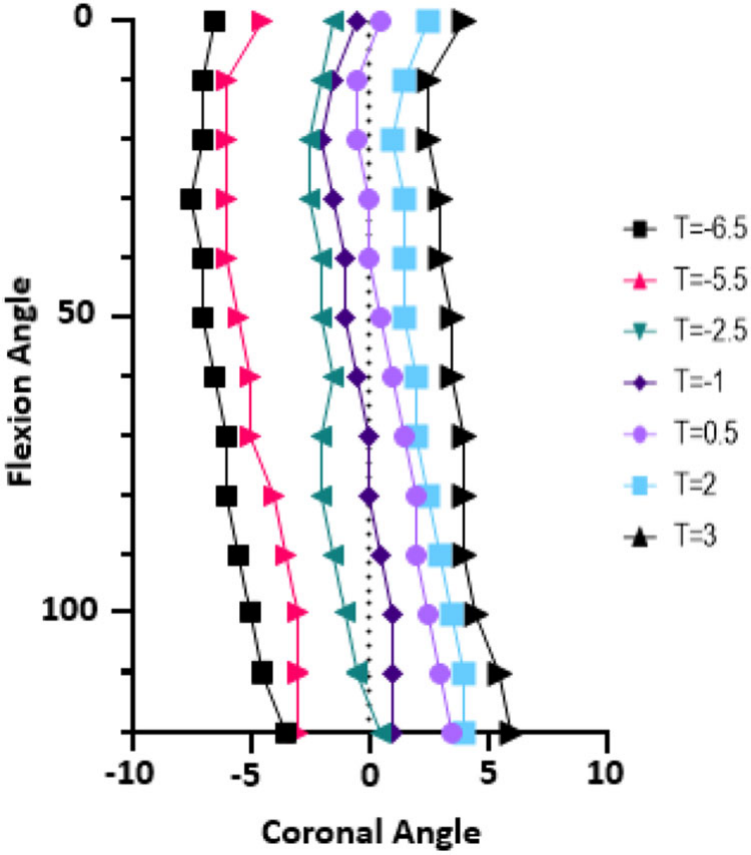
Curve Group 1 (F0TxR0) had a fixed distal femoral angle of 0° and femoral axial angle of 0°. The kinematic curves of the knee with seven different proximal tibial angles (PTAs) were recorded to examine the influence of the coronal tibial anatomy on the coronal kinematic curve. The same curve morphotype was reproduced regardless of the PTA. Note that different PTAs result in a different position of the curve relative to 0° but have no influence on the curve morphotype (*y*‐axis: knee flexion angle [°]; *x*‐axis: hip–knee–ankle angle [°], with negative values indicating varus).

**Figure 6:**
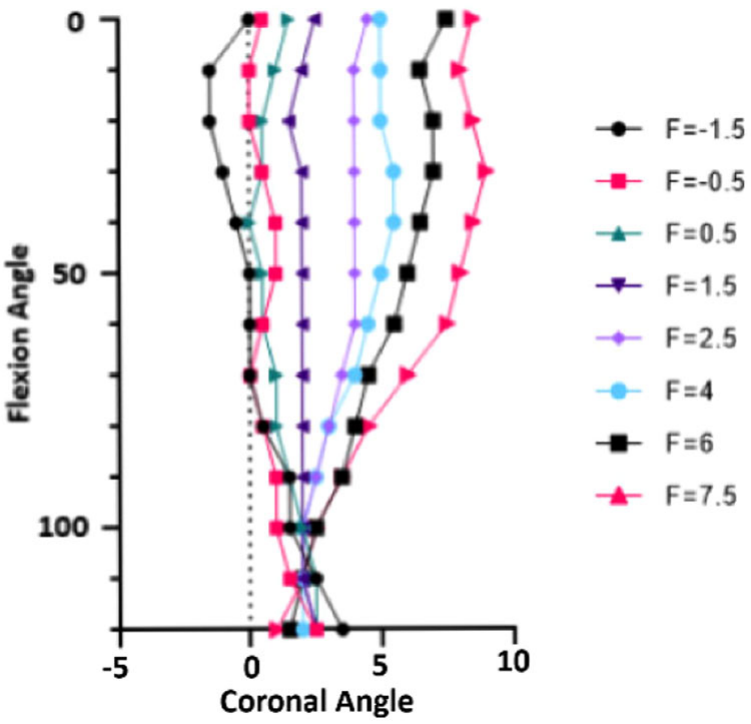
Curve Group 2 (FxT0R0) had a fixed proximal tibial angle of 0° and femoral axial angle of 0°. The kinematic curves of the knee with eight different distal femoral angles (DFAs) were recorded to examine the influence of the coronal femoral anatomy on the coronal knee kinematic curve. Note that the curve morphotype varies between the knees with the different DFAs. The effect of the DFA is greatest in extension and reduces as the knee flexes (*y*‐axis: knee flexion angle [°]; *x*‐axis: hip–knee–ankle angle [°], with negative values indicating varus).

**Figure 7:**
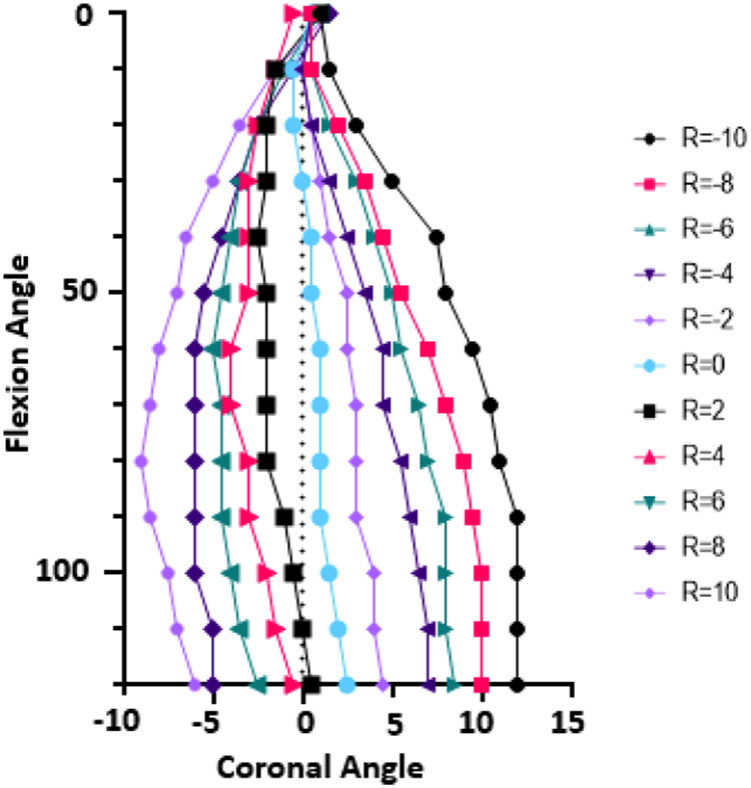
Curve Group 3 (F0T0Rx) had a fixed proximal tibial angle of 0° and distal femoral angle of 0°. The kinematic curves of the knee with 11 different femoral axial angles (FAAs) were recorded to examine the influence of the femoral torsion on the coronal knee kinematic curve. Note that the effect of changing the FAA is negligible in full extension, increases with flexion to a peak at 90° and then begins to reduce in deep flexion (*y*‐axis: knee flexion angle [°]; *x*‐axis: hip–knee–ankle angle [°], with negative values indicating varus).

Seventy kinematic curves were captured across seven groups with coordinates detailed in Table 1. Figure 5 illustrates the kinematic curves of Group 1, demonstrating the influence of the PTA on the kinematic curve. Figure 6 shows the kinematic curves of Group 2, demonstrating the effect of variations in DFA on the kinematic curve. Figure 7 presents the kinematic curves of Group 3, illustrating the impact of changes in the FAA on the kinematic curve.

Five curve morphotypes were identified including straight, drift, inverse drift, C‐shaped and inverse C‐shaped curves. No S‐shaped curves were identified

There was a significant difference in DFA across curve types (Kruskal–Wallis test, *H*(4) = 43.03, *p* < 0.0001; Table 3). After adjustment for multiple comparisons, there were significant differences (large *ε* ^2^) in the DFA between C‐shaped and inverse C curves, between C‐shaped and drift curves, between inverse C and inverse drift curves, between drift and inverse drift curves and between inverse drift and straight curves (Table 3).

**Table 3:** Mean (SD) distal femoral angle (DFA), proximal tibial angle (PTA) and femoral axial angle (FAA) for each curve type.

Curve type (number)	DFA mean (SD)	PTA mean (SD)	FAA mean (SD)
Straight (18)	1.31 (1.73)	−1.67 (2.64)	−0.89 (1.97)
Drift (17)	−0.41 (1.86)^a^	−3.32 (2.67)	−4.71 (3.80)^a^
Inverse drift (23)	4.63 (1.87)^a^ ^,^ b	−1.57 (2.78)	3.30 (4.42)^a^ ^,^ ^b^
C‐shaped (4)	4.63 (1.89)^b^	−1.63 (3.20)	−9.50 (1.00)^a^ ^,^ c
Inverse C‐shaped (8)	−0.75 (1.49)^c^	−3.13 (2.72)	7.25 (2.60)^a^ ^,^ ^b^ ^,^ d
*p*‐Value (Kruskal–Wallis)	<0.001	0.114	<0.001
Overall effect size estimate (ε2)	0.624	0.108	0.640

^a^ *p* < 0.05 versus straight;

^b^ *p* < 0.05 versus drift;

^c^ *p* < 0.05 versus inverse drift;

^d^ *p* < 0.05 versus C‐shaped.

There was a significant difference in FAA across curve types (Kruskal–Wallis test, *H*(4) = 44.13, *p* < 0.0001) (Table 3). After adjustment for multiple comparisons, there were significant differences (large *ε* ^2^) in the FAA between C‐shaped and inverse C curves, between C‐shaped and inverse drift curves, between inverse C and drift curves, between inverse C and straight curves and between drift and inverse drift curves.

PTA did not vary across curve types (Kruskal–Wallis test, *H*(4) = 8.20, *p* = 0.0844) (Table 3).

## DISCUSSION

The most important finding of this study was that the coronal plane kinematic knee curve morphology was determined by the distal femoral coronal and torsional anatomy (DFA and FAA). The coronal tibial anatomy influenced the position of the curve relative to the zero line but not the curve morphology. Importantly, all three parameters contributed to dynamic HKA. This study has provided additional evidence that the coronal plane knee alignment changes with flexion, and therefore, the dynamic knee alignment should best be described through a kinematic curve rather than a single HKA value.

Previously, Young et al. [20] used computer navigation to examine the curves of osteoarthritic knees undergoing TKAs. They examined the curves before and after TKA implantation and proposed a categorisation of kinematic curves involving seven types (Figure 2). After TKA, the kinematic curves changed towards neutral curves, but subtle elements of the initial curves remained. Saracco et al. [16] examined arthritic knees with computer navigation and concluded that limb alignment during extension was a poor predictor of knee alignment in flexion. Deep et al. [3] and Larrainzar‐Garijo et al. [7] examined arthritic knees undergoing TKA with computer navigation, noted that alignment changed in flexion for the majority of knees showing great variability, and each proposed a classification of the dynamic alignment. Neirynck et al. [12] examined arthritic knees undergoing TKA with computer navigation and found that only half of the knees show a straight curve (variation within +3°, −3°). The fact that the present study reproduced five of the seven curve morphotypes identified by Young et al. [20] lends credence to the validity of the sawbone model.

While these previous studies have shown that knee alignment changes in flexion, they did not directly clarify how variations in femoral or tibial anatomy contribute to specific curve patterns. As a result, these studies provide limited insight into the aetiology of curve morphology or guidance on how to reproduce or modify a specific curve through bone cuts during TKA. Our study, for the first time, systematically examined the relationship between femoral and tibial anatomy and the resulting curve morphotypes. The findings suggest that each anatomical parameter influences dynamic knee alignment in a distinct way across the flexion arc. Specifically, distal femoral anatomy appears most relevant in extension, while femoral torsion becomes critical around 90° of flexion, directly affecting whether the knee drifts into varus or valgus. In contrast, proximal tibial anatomy does not shape the curve morphology itself but rather shifts the curve's position relative to neutral. These insights highlight how variations in bone anatomy may help explain why patients present with different kinematic patterns and suggest which anatomical features surgeons might prioritise when aiming to reproduce or modify a curve during TKA.

### Implications in TKA

It is currently unclear whether a TKA should aim to reproduce the pre‐arthritic dynamic knee alignment or achieve a straight alignment. Regardless of the chosen strategy, this study has several potential implications for TKA. Since these implications are based on a bone model and have not yet been validated in cadaveric or clinical studies, they remain hypothetical and cannot be directly applied in practice. However, if these findings are confirmed in future clinical studies, the implications would be as follows:

Reproducing pre‐arthritic curve: If the aim is to reproduce the native curve, the pre‐arthritic distal and posterior femoral condylar joint lines must be reconstructed. This requires matched distal and posterior femoral resections accounting for chondral loss, which is the essence of a KATKA femoral technique [6, 11]. If the native curve is straight, KA would reproduce it, while MA with external femoral rotation would drive the HKA into varus during flexion.

Producing a straight neutral curve: Neutral distal femoral and proximal tibial resections will simplify the curve towards straight and neutral versions, reliably producing neutral HKA in full extension. Maintaining neutrality through flexion depends on native femoral torsion and femoral component position relative to the PCA. For C‐shaped or drift curves, external femoral rotation helps maintain neutrality, as these patterns are driven by internal torsion. Conversely, external rotation should be avoided in inverse drift or inverse C‐shaped curves. In simplest terms, if the curve shifts into varus during flexion, external rotation should be avoided; if it shifts into valgus, external rotation helps maintain neutrality.

Before adjustments, the preoperative curve type must be known. This can be acquired intraoperatively using navigation or robotics by recording a stressed HKA through flexion. Osteophytes are first removed on the diseased side, then the knee is flexed while tensioning the corresponding collateral ligament. For example, in medial OA, medial osteophytes are removed and the intact MCL tensioned against a largely normal lateral compartment, producing a reasonable approximation of the preoperative curve. Once the DFA and FAA are known, femoral implant coronal alignment and rotation can be customised to achieve the desired postoperative curve.

Altogether, this study delivers three clear messages regarding kinematic curve replication in TKA:

First, the pre‐arthritic DFA and FAA should not be altered if the native curve pre‐arthritic curve is to be reproduced.

Second, the same femoral component external rotation, as performed in MATKA [10] will not produce a straight neutral curve in all cases, and therefore, FAA should be adjusted individually if a straight curve is desired.

Third, reliably producing a neutral curve is only possible using computer navigation/robotics in TKA. In the case of conventional instrumentation, the surgeon has no information of the preoperative curve type and can therefore not adjust the femoral rotation accordingly.

### Curve morphotypes

Regarding possible curve variations, several types have been identified in previous studies, and various classifications have been proposed. In the current study, only five of the seven curve types described by Young et al.   [20] were observed (Figure 3). No S‐shaped or inverse S‐shaped curves were recorded. A possible explanation is that Young et al.  [20] examined only knees with severe osteoarthritis undergoing TKA. Osteoarthritic knees exhibit cartilage loss and, in some cases, bone loss, which can differentially affect the medial and lateral compartments, as well as distinct regions of the femur (distal femur or posterior condyles) and tibia (anterior or posterior). This asymmetric wear of the joint can result in greater variation in curves across different positions of the range of motion, which may not necessarily reflect the underlying bony anatomy. Additionally, our model lacked soft tissues, which can also contribute to more complex curve variations. Consequently, more complex curve patterns may arise.

In contrast, our model—while representing different combinations of DFA, FAA and PTA—was non‐arthritic. This suggests that only the five identified types may occur in non‐arthritic knees. This interpretation is supported by a comparison between our model and curves from non‐arthritic knees (unpublished data), which revealed the same five curve morphotypes. Furthermore, statistical analysis of DFA and curve morphotype yielded results similar to those found in vivo in non‐arthritic joints. We therefore consider the model to be at least partially validated in this regard.

### Influence of the outcomes of TKA

To date, there has been no analysis of the outcomes of TKA in terms of the influence of kinematic curve restoration or deviation from the pre‐arthritic curve. Therefore, it is unknown whether the outcomes of TKA will be optimised by restoring the pre‐arthritic kinematics or altering them to produce a straight neutral curve. Some curve types might be better restored, and others corrected. Further work is definitely required in this field.

### Dynamic knee alignment of healthy knees: Does it change in flexion?

The vast majority of the literature examining dynamic knee alignment included osteoarthritic knees undergoing TKA. This is easily understood, since there are ethical and practical reasons not allowing the usage of computer navigation or fluoroscopy on healthy knees. Two studies have reported on the kinematic curves of healthy knees. Using computer navigation, Siston et al. [17] examined the curves of non‐arthritic cadaver knees and TKAs. They reported that a consistent neutral alignment was not found either in healthy knees nor in TKAs with TKA restoring neutral alignment towards extension, but not in flexion. Varadarajan et al. [18] used fluoroscopy (repetitive x‐rays) to document the curves of non‐arthritic knees and reported that coronal knee alignment deviated from neutral in extension to varus in flexion. While these prior studies examined non‐arthritic knees, they did not examine the kinematic curves of each individual knee and reported only on average HKA values for all knees. For the first time, our study systematically examined the kinematic curves of ‘healthy’ tibiofemoral joints and confirmed that similar to arthritic knees, straight curves are not present in the majority of healthy knee joints.

## STRENGTHS AND LIMITATIONS

To date, there is minimal knowledge of the factors determining dynamic knee alignment. This study applied a unique method to provide an explanation of dynamic knee alignment and kinematic curve morphotypes. The current model had some definite strengths. The established accuracy of the technology utilised in the study was critical in ensuring the accuracy of model coordinates. The ranges of DFA (7.5° to −1.5°) and PTA (−6.5° to 3°) used in the study were representative of the values identified in normal subjects [1]. The curve groups included varus, valgus and joint obliquity outliers, which broadens the applicability of the results. The maintenance of condylar contact and the exclusion of large axial rotational movements during curve acquisition may serve as a simplified approximation of weight‐bearing conditions, though it does not fully replicate physiological loading. The choice of the PCA as the primary axial reference minimised potential differences in the virtual and actual rotation axis around which the tibia rotated [19]. However, the model has significant limitations. Being based solely on condylar contact in a sawbone model, it cannot account for the influence of soft tissues, muscle forces or complex joint kinematics such as differential rollback and medial pivot, all of which are likely to affect curve morphology. Furthermore, the experimental setup defined the femoral cylindrical axis as the flexion–extension axis, which may have introduced some bias towards femoral parameters influencing curve morphology. Additionally, each configuration was registered and measured only once; therefore, we did not perform repeated registrations or calculate intraobserver variability. Finally, all measurements were conducted under non‐weight‐bearing conditions, and therefore, the dynamic coronal plane alignment under physiological weight‐bearing remains to be investigated. These shortcomings are critical and limit the direct applicability of our findings. Future studies using cadaveric models would be essential to address these deficiencies and validate the results under physiologic conditions.

## CONCLUSIONS

Coronal plane knee alignment changes with flexion, and therefore, the dynamic knee alignment is best described through a kinematic curve of HKA rather than a single HKA value. Distal femoral coronal and torsional anatomy determines the morphology of the kinematic curve, while tibial anatomy influences its position. Future studies should validate the current novel results in cadaveric or clinical settings and explore their implications for TKA.

## AUTHOR CONTRIBUTIONS

Petros Ismailidis participated in the measurements, took part in the interpretation of the data and wrote manuscript. Peter McEwen conceived the study, performed the measurements and the statistical analysis, participated in the interpretation of the data and the writing of the manuscript. Kenji Doma and Anthony Leicht performed the statistical analysis and revised the manuscript. All authors read and approved the final manuscript. All authors had full access to all data and take responsibility for the integrity of the data and the accuracy of the data analysis.

## CONFLICT OF INTEREST STATEMENT

The authors declare no conflicts of interest.

## ETHICS STATEMENT

The study required no ethics approval. No human participants were involved.

## Data Availability

The data that support the findings of this study are available from the corresponding author upon reasonable request.
